# Surface treatment and post-processing of metallic bio-implants: enhancing performance and biocompatibility

**DOI:** 10.3389/fbioe.2026.1755334

**Published:** 2026-04-10

**Authors:** C. Uvanarayanan, P. Suya Prem Anand, Geetha Manivasagam, Prudvireddy Paresi, Jesuarockiam Naveen, B. Ranjeet Kumar

**Affiliations:** 1 School of Mechanical Engineering, Vellore Institute of Technology, Vellore, India; 2 Center for Biomaterials, Cellular and Molecular Theranostics, Vellore Institute of Technology, Vellore, India; 3 Engineering Technology and Science, Higher Colleges of Technology, Bani Yas A, Abu Dhabi, United Arab Emirates; 4 Department of Mechanical Engineering, National Institute of Technology, Kurukshetra, India

**Keywords:** bioimplants, ion implantation, laser shock peening, surface texture, surface treatment

## Abstract

This review investigates the critical role of surface treatment and post-processing techniques in enhancing the performance and biocompatibility of metal bio-implants. The paper addresses the challenges posed by the significant difference in Young’s modulus between natural bone (15–45 GPa) and metal alloys (110–240 GPa), which leads to stress shielding effects and potential toxic ion release. The review first details various surface coating methods, including ion implantation and anodization, highlighting their ability to improve tribological resistance, corrosion resistance, and biocompatibility. The detailed analysis gives surface modification techniques, such as laser shock peening, Nitrogen Plasma Immersion Ion Implantation (NP-III), and anodization, which are used to enhance titanium implant properties, such as increasing surface hardness, promoting tissue growth, and creating a bio-active oxide layer. Furthermore, the paper explores post-processing methods such as laser shock peening (LSP) and surface texturing, which are crucial for modifying the surface topography and microstructural properties of implants. It also discusses techniques, particularly laser-based texturing, to reduce friction and wear while inducing beneficial compressive residual stress. The review concludes by emphasizing that a tailored approach to surface modification and post-processing is essential for developing safe and effective bio-implants for a wide range of applications, from bone fixation to load-bearing joints.

## 1 Introduction

**FIGURE 1:**
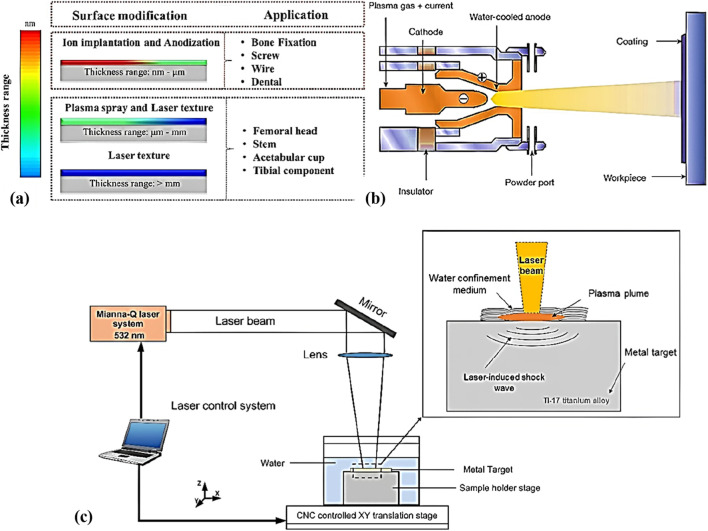
Surface treatments for different biomedical applications, **(a)** Impact of surface treatment on thickness, **(b)** Plasma spray technique (Heimann, 2023), and **(c)** Schematic diagram of laser shock peening (Jiao et al., 2019).

The cell interaction, tissue growth, and response largely depend on the surface properties of the metal alloys, such as hardness, wettability, and surface roughness (Ratner and Castner, 2020). While implanting the metal alloys in the human body, a chronic biological response usually occurs around the implanted materials. The load-bearing joints (hip and knee) require a combination of high strength, ductility, and tribological resistance to avoid the failure and degradation of the material (Ralls et al., 2020). The issue with the metal alloys is a leaching of high-concentration nickel, chromium, and cobalt ions, which affects the immune system and leads to inflammation. Additionally, stress shielding effects on metal implants often accelerate the corrosion and wear activities (Geetha et al., 2009). To overcome the above-discussed challenges in the metal alloys, the surface modification technique is essentially required. Surface modification techniques such as physical and chemical vapour deposition are used for biomedical applications (Zhu et al., 2021). Precisely, surface roughness plays a major role in the Osseointegration process, where it improves the adhesive strength between the tissue and substrate. However, the higher roughness in the materials releases the toxic elements from the material during the long-term implantation process. To overcome these, the coating technique handles coating the bio ceramic particles on the surface using the Plasma Spray Technique (PST). Similar to the PST, the anodization process modifies the surface of the material by forming the nanostructured oxide layer, which helps to improve the tissue growth and enhance the corrosion resistance. Meanwhile, coating technique required the post annealing process to over the adhesive strength, and coating defects challenges. This review also explores the suitable surface modification technique based on the demands of targeted applications by avoiding the additional post processing technique. Figure 1a shows the surface modification techniques that are used to modify the surface from nano to micro scale, micro to millimeter scale, and greater than a millimeter for different biomedical applications.

### 1.1 Coatings of bio-implants

The coating is one of the surface modification techniques used to modify the surface and improve the biocompatibility of the materials. Physical and chemical surface modification methods, such as anodization, plasma spraying, laser texturing, and ion implantation, are used in the biomedical industries (Priyadarshini et al., 2019) (Table 1). All these methods are essentially used for manufacturing biomaterials to achieve maximum biocompatible properties. In recent years, hybrid approaches combining coatings with nanoscale texturing have also gained attention because they simultaneously improve corrosion resistance and promote osteointegration.

**TABLE 1:** List of surface treatments performed on different materials.

S.NO	Surface treatment	Material used	Treatment condition	Applications	References
1	Ion implantation	Substrate material: Mg alloys.(WE43) Coated material: Fe, Ti, Zn, and Zr ions	Energy/KeV: 50 Doses/ions cm^−2^: 1× 10^16^	Bioimplant	Li Z. et al. (2025)
2		Substrate material: Iron (Fe) Coated material: Zn	Energy/KeV: (45/60/45) Doses/10^16^ions cm^−2^: Zn (10/10/20)	Bioimplant	Wang et al. (2025)
3		Substrate material: Silicon substrates and titanium alloy-based substrates Coated material: Zr/Nb ion	Voltage (kV): 10. Magnetic bias (Vs.): 10; 10 Pulse frequency (Hz): 6	Bio-implant coating materials	Gao et al. (2023)
4	Anodization	Substrate material: Titanium Coated material: Titanium oxide (nanotubes)	Reaction conducted at 15 °C, 180 V and 3 h duration	Dental implants, drug delivery	Li et al. (2020)
5		Substrate material: Titanium Coated material: Titanium oxide (nanotubes)	Heat treatment between (450 °C–500 °C), Transfer amorphous to crystalline structure	Nanosensors, biomaterials, controlled release of ions/drugs	Mansoorianfar et al. (2021)
6		Substrate material: Magnesium alloy(AZ 31) Coated material: Calcium phosphate	At 20 V, reduce the degradation rate	Biomedical devices (biodegradable)	Simi et al. (2025)
7	Plasma spray technique	Substrate material: Metallic implants (Co-Cr, SS, and Ti) Coated material: Hydroxyapatite	Spraying HAP at high temperatures affects the material surface	Knee replacement, hip implant, and long bone fracture	Ratha et al. (2021)
8		Substrate material: Titanium Coated material: Hydroxyapatite	Preheating the titanium sample improves the attainment of a uniform nanostructure. Temperature range: 200°–1000 °C	Bioimplants	Fomin et al. (2017)
9	Laser texturing	Surface texture: Dimple and line	Discussed the influence of laser process parameters	Bioimplants, piston cylinder assembly and cutting tools	Prasad et al. (2022)
10		Substrate: Ceramic Surface texture: Dimple pattern	Dimple diameter: 15 μm, dimple density: 15%	Hip joints arthroplasty	Roy et al. (2015)
11		Microtexture: Interface between silicon nitride and hardened steel Surface texture: dimple structure	5%–20% density Depth – 100, 150, and 200 µm 100 µm dimple outperforms other conditions	Automotive engine	Wakuda et al. (2003)
12		Cobalt chromium alloy Surface texture: Dimple, line, net, and surface Interacting surface: Ultra-high molecular weight polyethylene	Depth range: 2.8–3.6 µm	Hip implant joints (acetabular cup)	Alvarez-Vera et al. (2021)

#### 1.1.1 Ion implantation

Ion implantation is a technique used to modify the surface of biomaterials for improving tribological resistance and bio-compatibility properties (Wang W. et al., 2021). Initially, the biomaterials were directly implanted in the human body by reducing surface roughness. However, improving the surface quality by reducing the surface roughness is not perfectly suited for implantation because in the human body, the metal alloys react with the protein and buffered solution, leading to the leaching of highly concentrated ion elements such as chromium and nickel (Eliaz, 2019). Therefore, to improve the tribological resistance and biocompatibility, high-energy ions are used to modify the surface quality of the material by using ion implantation (Viswanathan et al., 2018).

Ti6Al4V is one of the best alloys among the Titanium alloys, which is recommended for the hip stem due to its multifunctional properties, such as high strength and corrosion resistance (Suresh et al., 2021). However, the presence of vanadium (V) in Ti6Al4V causes a biological risk to the patient (Chandra et al., 2010; Suresh et al., 2021). To overcome this limitation, researchers developed Ti6Al7Nb alloys, where V was substituted with Nb. Although this improved the biological safety, the initial response in terms of biocompatibility was relatively low (Huang et al., 2013; Huang et al., 2021). Enhanced the biocompatibility and corrosion resistance of the Ti6Al7Nb by using Nitrogen Plasma Immersion Ion Implantation (NP-III). Two different voltages, 5 and 20 kV, are used with 16 min of implantation time and a width of 10 μs. In addition, an untreated sample is used for the comparative studies. Later, untreated and ion-implanted Ti6Al7Nb samples are carried out for corrosion, *in vitro*, and *in vivo* tests. The corrosion test is performed at the scan rate of 1 mV/s from −0.2–2.0 V. In addition, human bone marrow mesenchymal stem cells and adult male pigs (18–20 kg) are used for the *in vitro* and *in vivo* studies. The potentiodynamic polarization curve shows that the 20 kV ion-implanted sample has a higher corrosion resistance (1109 mV), whereas the untreated sample’s corrosion resistance is −375 mV, and for 5 kV is 830 mV. Similarly, high voltage ion energy (20 kV) attained high positive results in the *in vitro* and *in vivo* studies, where high energy voltage enhances tissue growth. In addition, the high voltage from the NP-III technique helps to improve the hardness of the coating, where coated samples for 20 kV attained 14.3 GPa maximum hardness at 15 nm depth.

Recent work has also expanded the application of ion implantation to bioresorbable and polymer-based implants. Dong et al. (2021) implanted manganese (Mn) on the magnesium surface to improve the corrosion resistance and avoid premature failures. A dose of 2 × 10^16^ cm^-2^ Mn ions was implanted on the surface by using a 70 V arc at 25 Hz frequency. Later, treated and controlled samples were immersed in a Hank’s solution to estimate the corrosion resistance. The corrosion test was performed at a scanning rate of 1 mV/s from −0.02 to −0.1 V. Finally, the Mn-implanted magnesium samples attained significantly higher corrosion resistance than the untreated samples. On the other hand, NP-III was coated on the PEEK samples to enhance tissue growth in the scaffold application. Initially, mesh-type PEEK samples were fabricated with the help of the FDM technique. Later, the materials were carried out to the post-processing technique for NP-IIM. The nitrogen ions were induced on the material at pressures of 0.467 and 0.934 mbar for a 20-min treatment. Finally, post-treated and controlled samples were tested in both *in vitro* and *in vivo*. The post-treated samples exhibited notable tissue growth in the biological test and showed a good response in the cell adhesion (Kruse et al., 2022). Li L. et al. (2025) investigated the effect of ion implantation (Fe, Ti, Zn, and Zr ions) on the corrosion resistance and biocompatibility behaviour of biodegradable Mg alloys (WE43). It has been found that Fe and Zn ions were not suitable as an implanting ion, since they do not reduce the degradation rate. On the other hand, Zr- WE43 exhibited very less CV (cell viability). Hence, Ti-WE43 can be considered the best choice with excellent biocompatibility and corrosion resistance. However, the Zn ions are highly essential for the endothelial cell integrity, where they maintain the vascular tone and regulate inflammation. Hence, Wang et al. (2025) incorporated Zn into degradable iron through ion implantation and performed the *in vivo* studies. The three gradient structure layers observed on the surface of the Zn/Fe, where the top surface observed the presence of multiphase oxide, the subsurface found enrichment of Zn, and the innermost layer observed high density dislocation structure. These three gradient layers enhance the long term corrosion stability, and the result observed that Zn/Fe exhibited accelerated corrosion behavior during 120 days of *in vitro* immersion experiments. Gao et al. (2023) investigated the effect of co-implanting Zr and Nb ions into ZrO2 film through magnetron sputtering and ion implantation techniques. It has been found that varying ion doses (50,60 and 70 min) greatly affect the performance. Zr/Nb-ZrO2 (60 min) samples showed superior corrosion resistance compared to other samples. Meanwhile, the challenges observed in the ion implantation are the usage of high ion doses, which leads to thermal instability, amorphization, and atom displacement. In addition, the Zn implantation in the magnesium alloy leads to galvanic effects and degrades the material highly in the biological fluids (Wu et al., 2011). Further, it causes inflammation or toxicity to the patient.

#### 1.1.2 Anodization

**FIGURE 2:**
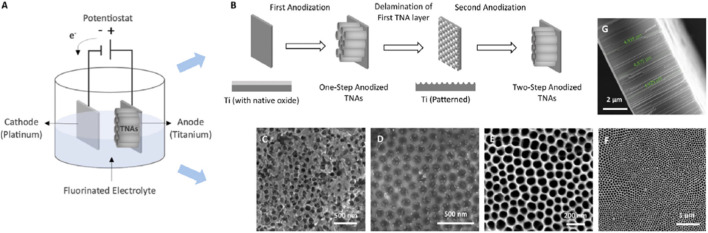
Schematic diagram and two-step anodization method. **(A)** Schematic diagram, **(B)** two-step anodization method and nanotube formation, and **(C–G)** top and cross section of the nanotubes (Opolot et al., 2024).

In an earlier stage of biomedical applications, the metal alloys were primarily used as an inert material, and there was no interaction or response with the surrounding tissue of a host body. To address this limitation, the bio-inert materials were converted into bio-active ones by modifying their surfaces with coatings such as hydroxyapatite and oxide layer (Natarajan, 2016). Anodization is a cost-effective surface modification technique that produces an oxide layer at the nano-level for enhancing adhesion strength and tissue growth. This method leads to the form of two types of surface modifications, such as the oxide layer and nano-porous structure layers (Park et al., 2017; Zwilling et al., 1999). Figure 2. Represent the schematic diagram of the two-step anodization method.

The presence of fluoride ions in the electrolytes facilitates the formation of a nano-porous structure within the oxide layer. The resulting nanotube lengths typically vary from 300 nm 50 μm and diameters vary between 30 nm and 50 nm, depending on the applied voltage, electrolytes, and time during the anodization process (Aw et al., 2011; Yao and Webster, 2009). In the anodization process, only the conductive materials are applicable for the surface modification technique because this process is done with the help of a direct current. Titanium and its alloys are commonly used in the anodization process because titanium possesses a low Young’s modulus among metallic alloys and is highly recommended for bone fixation, screw, and stems (Niinomi and Nakai, 2011).

Involving the titanium in the anodization process leads to modifying the surface of the material by forming the TiO_2_ nano-porous oxide layer, and it influences the interaction of the surrounding tissue with the host body (Li et al., 2020). Meanwhile, materials converted from bio-inert to bio-active experience difficulties in tissue growth. To overcome this, the tube length and diameters are increased with the assistance of high voltage and electrolytes (Qadir et al., 2020). The TiO_2_ nanotubes developed in this process are generally amorphous in structure. In biomedical applications, the crystal structure is favored because the amorphous state is less active than the crystal structure when it comes to biocompatible properties (Bashirom et al., 2022; Mansoorianfar et al., 2021). Therefore, the temperature for heat treatment, 450 °C–500 °C, is predominantly used for converting TiO_2_ from amorphous to a crystalline form. Another interesting fact found is that heat treatment also supports improving the nanotube length and diameters, but heat treatment above 600 °C collapses the nanotube dimensions (Jarosz et al., 2015).

Decha-umphai et al. (2021) estimated the adhesive strength of anodized TiO_2_ layers on SLM-printed Ti6Al4V alloys. In addition, heat treatment and annealing were done to enhance the adhesion strength and attain TiO_2_ in the crystal structure. Anodization was performed on three sets of samples. In set 1, polished as-printed samples were directly used for the anodization process without any pre-heat treatment process. In set 2, the anodized samples were annealed at 500 °C for 2 h. Finally, in set 3, the printed samples were heat-treated at 950 °C for 2 h before the anodization process, followed by annealing under the same conditions as set 1. A nano-scratch test was conducted using a maximum load of 30 N, a scratch length of 1 mm, and a velocity of 6.3 mm/s. The results showed that heat-treated and annealed samples from set 3 attained high adhesion strength (scratch distance 151.52 μm) compared to others. Moreover, nature motivates the development of a bamboo-type nanotube structure (nanotube + compact layer + nanotube) in the anodization process, which results in the predominant growth of nanotubes (Zhang et al., 2019).

A double oxide layer is formed on titanium when the substrate material is anodized with different ions such as phosphate, sulfate, and iodide. The titanium oxide formed consists of a dual-layer structure with a robust porous structure developed as the outer layer and a stable oxide layer formed beneath the porous layer as a dense layer. The result indicates that anodized samples show better corrosion resistance compared to the polished titanium (Mehta et al., 2025). A double-layer duplex coating is performed on the magnesium alloy (AZ31) with anodic oxide initially followed by calcium phosphate to reduce the degradation rate of magnesium. The sample exhibits a uniform coating of calcium phosphate on the oxide layer at 20 V using the electrodeposition method. Finally, the surface morphology indicated the complete sealing of the oxide layer with calcium phosphate to control the degradation rate of magnesium (Simi et al., 2025). Titanium β phase alloy is frequently used as a biomaterial due to its low Young’s modulus and high corrosion resistance. But the surface properties are not sufficient to support the biomaterial behavior. Therefore, an oxide layer is formed on the surface of the Tiβ phase alloy by using the anodization method under different potentials. The elasticity of the treated sample is improved to 83 GPa compared to the untreated sample. The treated sample exhibits optimum conditions at 200 V (30 min) to improve wear property, Ra value, and surface morphology (Şenaslan et al., 2023).

#### 1.1.3 Plasma spray technique

The Plasma Spray Technique (PST) is a physical surface modification technique followed by ion implantation and laser cladding for biomedical applications. In this process, materials such as metals, ceramics, and polymers are coated onto a material surface by converting it into a molten or semi-molten state with the help of direct current during experiments (Natarajan, 2016). Figure 1B shows the processing diagram of the plasma spray technique.

PST is primarily used to improve the Osseo-integration of the implant materials by spraying the hydroxyapatite (HAp) particles at high temperatures (Ratha et al., 2021). However, coating the HAp at high temperatures can lead to structural changes and degrade a material’s surface. Simultaneously, it affects the material properties, peel of the coated surface, and attains low adhesion strength between the material and tissue (Fomin et al., 2017; Khor et al., 2004). To improve the adhesion strength, the materials are pre-heated at temperatures between 200 °C and 1000 °C. Meanwhile, pre-processing (heat treatment) of the samples at 400 °C–600 °C influences the attainment of a uniform nano-structure with high hardness (0.9–1.2 GPa) and Young’s modulus (7–16 GPa) (Fomin et al., 2017). However, the adhesion strength of the coated samples is not concluded with proper data. To improve the mechanical properties, bio-compatibility, and antibacterial properties, the post-heat treatment is carried out at 500 °C and 600 °C for 3 h. The obtained results favoured the 500 °C heat-treated samples, whereas the 600 °C heat-treated samples were found to be toxic while testing under pre-osteoblast (MC3T3-E1) cells and against biomedical applications. However, post-heat treatment at 600 °C attained good mechanical properties and anti-bacterial properties (Ullah et al., 2020).

To further improve coating performance, researchers have developed composite plasma coatings (HAp - Carbon nanotubes). For example, coating the HAp reinforced with 4% carbon nanotube powder achieved higher mechanical and tribological properties than the HAp-coated samples (Lahiri et al., 2011). On the other hand, drugs such as curcumin and vitamin K2 are coated on the Hap-coated surface to improve the Osseo-integration of the implant material. The drug-loaded samples are tested in both *in vitro* and *in vivo* studies. Due to the presence of Hap with these drugs, the cell survival rate is nearly 92%–95% higher compared to untreated samples. Despite its advantages, PST generally increases production cost compared to the anodized samples. Therefore, PST-coated materials are highly recommended for fabricating acetabular shells. Otsuka et al. (2021) modified the acetabular shell by coating a Hap on the surface with the help of PST. This study highly focused on solving the non-inflectional loosening problem, which is caused by the load-bearing activities of patients in day-to-day life. Before estimating the wear behavior, the samples are coated by using these process parameters: voltage 68 V, current 500 amp, spraying rate 120 mm/s, and feed rate 20 g/min. Later, the materials are subjected to a wear test with the help of a simulator, and the test is conducted at a load of 2.35 KN, frequency of 1–5 Hz, × 10^6 cycles. Finally, the obtained result shows that the coated thickness of 150 μm surface found with delamination, which causes aseptic loosening for the patient. In the future, improving the coating thickness might enhance the tribological properties and reduce the causes for loosening. From the observation of the above-discussed coating method, post-annealing is essentially required for the ion implantation and anodization technique, which helps avoid the ion implantation damage and changes the amorphous structure into a crystalline one. For the ion implantation, the temperature might change based on the ions, which is used for the implantation. For example, the N ion and Al ion required above 1300 °C and 1500 °C (Ge et al., 2021). Meanwhile, the LSP method is used for various applications to enhance the surface hardness and corrosion resistance. Further, LSP is aided as a post-processing technique for the Additive manufacturing parts, and no additional post-annealing is required for the LSP-treated sample. The LSP and laser texture methods are discussed in the sections below.

## 2 Post-processing of bioimplants

The performance of AM medical implants strongly relies on their surface topography and microstructural properties. These characteristics directly influence the interaction between the implant and surrounding tissue. Optimised process parameters during printing and post-processing would enhance the biological performance of customized implants. Design for AM (DfAM) gives more freedom to achieve tailored structural and microstructural properties, with the minimum possible weight. Mechanical properties and topography of implants would be altered by standard post-processing techniques, e.g., sandblasting, hot isostatic pressing, and ultrasonic abrasive polishing (Roudnicka et al., 2019). Due to improper fusion and gas entrapment, residual pores are generated. Wu et al. (2017) reported that fatigue resistance of the SLM printed components is enhanced by reducing the residual pores by the Hot Isostatic Pressing (HIP). It is difficult to perform finishing operations because AM parts are complex with manifold internal features. Wang et al. (2019) studied the effect of ultrasonic abrasive polishing on complicated components printed by AM. Cosma et al. (2017) investigated the influence of three combined post-processing methods, i.e., alumina carborundum polishing, sandblasting, and ultrasonication, on implants manufactured by SLMHI. This study was focused on enhancing the surface properties of the complex contact area between the model material and support structures Hashmi et al. (2023). Reviewed the various surface modification techniques for biomedical applications. Among the surface modifications, a unique process mentioned as a hybrid-based process, where the additive printed parts are surface treated using the laser shock peening, ultrasonic peening, plused laser deposition, and friction stir processing. In orthopaedic application (load bearing joints), the LSP methods will be preferable method, which helps to enhance the wear resistance, hardness, and reduce the co-efficient of the friction. Further, the laser texture methods help to form micro grooves, microstructure, and micropumps. As a results, the biological response and mechanical properties are enhanced. In addition, magnesium-based alloys are aided for the biomedical application and preferrable for the temporary implant as a bone fixation plate, and screw to avoid the secondary surgery. While approaching the magnesium alloy, materials will degrade and biocompatible, which does not affect the human body. However, the magnesium alloy will degrade faster before the bone healing period. To minimize the degradation rate, the surface modification technique is essentially required (Singh et al., 2024). This process will be discussed in below.

### 2.1 Laser shock peening of bio-material surfaces

**FIGURE 3:**
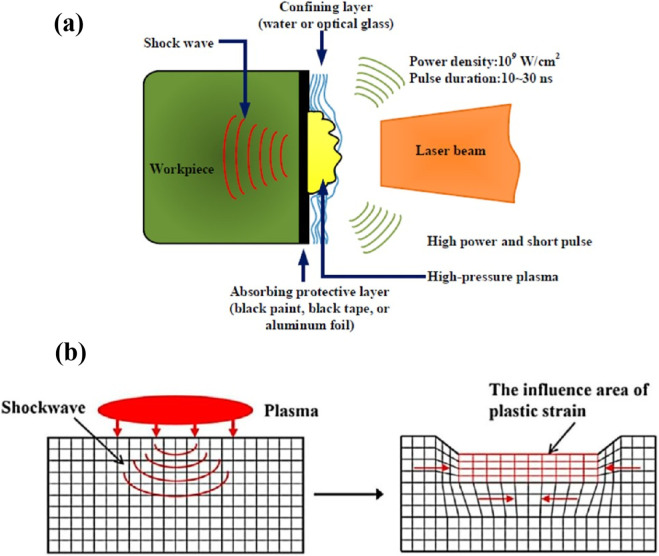
**(a,b)** the schematic diagram of laser shock peening and plastic strain area (Cao et al., 2024).

The tribological and bio-compatibility properties of implants strongly depend on the surface quality of the materials (Zhao et al., 2021). The laser shock peening (LSP) technique is essentially used to enhance the surface functionalities for achieving the maximum tribological resistance and bio-compatibility properties in biomedical applications (Cao et al., 2024; Shivakoti et al., 2021). Materials such as metals, ceramics, and polymer-based materials can be treated with the LSP technique for both temporary and permanent orthopedic implants (Shukla et al., 2016). According to the metallurgy perspective, the LSP is a cold working process, which can induce compressive residual stress with high dislocation densities (Figures 3a,b). Shows the mechanism of the LSP and of the shock wave, which deforms the material surface at a high strain rate (Cao et al., 2024). Thus, it helps to enhance the hardness, and it can achieve the high strain rate (10^6^/s – 10^7^/s) (Gujba and Medraj, 2014; Peyre et al., 2012).

Based on the LSP process parameters, the depth of the compressive stress could vary, and it may achieve up to the mm range. Additionally, the mechanical performance of the LSP-treated material is outstanding in fatigue, wear, and corrosion resistance (Gupta et al., 2019). LSP technique used as a post-processing for the AM fabricated components, where the LSP is used as a tool and re-engineered for relieving the thermal stress and changing the microstructure and chemical composition of the surface. In addition, LSP was used to fabricate the surface layer, which consists of an amorphous and nanocrystalline layer on the TC 11 titanium alloy (Kalentics et al., 2017; Luo et al., 2018). Studies performed simulation analysis using the finite element method and compared with the experimental results, where the LSP method found 287% in the fatigue life (Adu-Gyamfi et al., 2018). On the other hand, the LSP pulse direction was changed from parallel to perpendicular towards the fatigue load direction. Thus, the fatigue life was enhanced from 166% to 471% (Correa et al., 2015).

Apart from the LSP, other surface modification techniques such as machining, UV-lithography, deep reactive ion etching, and shot peening are also proposed (Wang Z. et al., 2021). However, LSP offers a distinct advantage because it combines the benefits of mechanical strengthening with minimal thermal effects, which helps preserve the intrinsic properties of the material. Figure 1C shows the schematic diagram of laser shock peening. In this process, an Nd: YAG laser penetrates the material surface to a depth of about 1–2 mm and induces the compressive residual stress (Velu et al., 2021), whereas conventional machining also induces compressive residual stress but only at shallow depths of 0.05–0.2 mm (Iida et al., 1995).

In industry practice, the LSP method is recommended for surface modification because it enhances mechanical performance at a low production cost (Niinomi, 2003). Therefore, the LSP technique is mostly preferred for biomedical and high-temperature applications like aerospace (Praveenkumar et al., 2023). However, researchers concentrated on the LSP technique for improving the tribological resistance of materials by texturing different patterns, such as dimple and line texturing (Prasad et al., 2022). LSP is used to penetrate the surface depth at the micro to mm level, and the depth level is controlled with the help of laser power. The peened surface area consistently shows higher hardness compared to untreated areas due to the compressive residual stresses induced during peening. Micro-level depth can also be performed with the help of LSP by optimizing the laser power, and the resulting changes are typically characterized using surface topography analysis. The material surface is hardened because of the stress/strain rate and compressive residual stress effects. Finally, the peened materials are sufficiently used for the biomedical and aerospace industries, where the materials are subjected to sliding and rolling motions (Guo and Caslaru, 2011).

### 2.2 Surface texturing to reduce the friction

As mentioned earlier, the LSP technique significantly enhances the tribological resistance of the materials. In recent years, surface texturing like dimple patterns has been used to improve the tribological properties. Similarly, the titanium alloy is coated with a multilayer of chitosan, HA, ZnO, and TiO_2_ to improve the biocompatibility and osseointegration. Further, the coated titanium alloy is laser textured with dimple patterns at different distances, such as 100 μm, 150 μm, and 200 µm. The result exhibited a better cell adhesion for a dimple distance of 100 μm at a dimple density of 5%–20% (Roy et al., 2015). Microtexturing the surface with small dimples will reduce the friction at the contact interfaces between silicon nitride ceramic and hardened steel. The small dimples will act as a fluid reservoir to retain the lubricant film and utilise it in the heat-generated area. The pin-on-disk experiment is carried out to analyze the effects of dimple size, density, and geometry on the coefficient of friction between the contact surfaces. The results indicated that the effects of size and density of the dimple affect the friction but not the shape (Wakuda et al., 2003).

Alvarez-Vera et al. (2021) textured the dimple, line, net, and surface patterns on the Co-Cr alloy to estimate the tribological resistance of the material. The parameters such as laser power 6000 W and scanning speed 1 mm/s are used for texturing the line, net, and surface area, and the same power with 2 mm/s scanning speed is used for dimple texture. Later, the pin-on-disk instrument is used for estimating the wear performance of the textured sample. The ultra-high molecular weight polyethylene acts as a pin, and textured samples are fixed as a disc in the instrument. The load of 10 N was applied with 7 different rotational speeds (50–200) for 1000 cycles. Total experiments are done in a lubricant environment (fetal bovine serum) at a temperature of 37 °C. The XRD and topography results found that the textured samples were obtained in fcc and hcp phase structures with dendrites. Therefore, textured samples attained a lower coefficient of friction. Among all the texture samples, the dimple texture was found with a lower friction rate of 0.1 compared with line (0.2), surface (0.25), and net (1.75).

Nakai et al. (2021) textured the dimple pattern on the β alloy (Ti-29Nb-13Ta-4.6Zr) and ɑ +β alloy (Ti-6Al-4V) for estimating the wear behavior of the material for dental applications. The process parameters used for texturing the dimple pattern are: laser power 30 W, hatch spacing of 50–200 μm, pulse width of 8 ps, and irradiation time of 2–6 ms. During the experiment, the dimple dimension was 45 μm, constantly fixed for all the samples. The ball-on-disc test is conducted on the textured and control samples to analyze the frictional wear behaviour. Here, a load of 2.94 N, a sliding speed of 60 rpm, and 6000 cycles are used for performing the wear test. Results showed that textures with 20 μm depth and 200 μm hatch spacing attained high wear resistance. In addition, β phase alloy shows better wear properties than ɑ +β alloy.

## 3 Surface modification challenges

**FIGURE 4:**
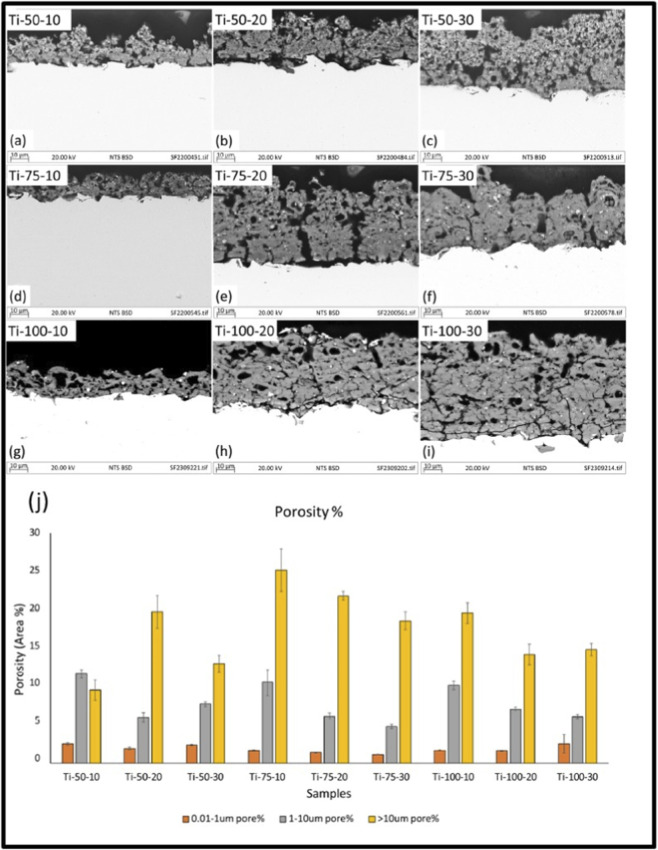
The back–scattered scanning electron microscopy image of the coated sample. **(a–i)** cross-sectional image, and **(j)** the porosity distribution (Mittal G et al., 2023).

Surface roughness is a key factor influencing the Osseointegration process in the biomaterial. The laser-based and plasma spray techniques treated samples have a desirable surface roughness at a micro-level for enhancing tissue growth and adhesion on a material surface. Further, the surface roughness is altered by the process parameters, which are used in the coating and laser treatment. In the plasma spray technique, the surface roughness was varied from 7.9 to 9. 5 μm at a spray distance of 70–130 mm. Meanwhile, scanning electron microscope images found the presence of semi-melted particles along with the crack propagation in the coating R.A. (Abbas et al., 2021). As mentioned earlier, the higher roughness leads to an enhancement of the current density and starts to degrade. The current density of surface roughness ∼9.6 μm was found at 1.28 × 10^−6^ A cm^-2^, whereas surface roughness ∼39 μm resulted with the current density of 3.71 10^−5^ A cm^-2^ (Wang, 2023). Therefore, the bio ceramic (Hydroxyapatite) coating might be a choice for the absence of toxic ion elements leaching from the materials (Nadian et al., 2025). Further, the high roughness bio-ceramic coating may enhance the Osseointegration process and help to overcome the above-discussed toxic elements challenges. Further challenge in the PST is the adhesive strength between the substrate and coating layer. Similar to the surface roughness optimization, the adhesive strength between the substrate and coating layer is controlled by the coating parameters. The particle’s velocity is a major parameter to coat the particle on the substrate, and it is managed by the gas flow rate, where a high gas flow rate reduces the plasma temperature. This leads to the formation of porosity, semi-melted powder particles, and cracks in the coated layer. A reported study found that at gas flow rates of 35 and 40 lpm, the adhesive strength was increased from 31 MPa to 41 MPa. Further increasing the gas flow at 45 lpm, the adhesive strength starts to decrease (Swain et al., 2022). Mittal et al. (2023) Coated the TiO_2_ nano particles on the substrate by varying the feedstock flow rate from 10 to 30 mL/min and stand off distance 50–100 mm with an interval of 25 mm. Figures 4a–j shows the porosity defects and porosity % while varying the coating parameters. The cross-sectional image shows the presence of porosity in the coated region, as shown in Figures 4a–i. The porosity observed was similar in all the conditions of the coating parameters. However, the coarse porosity was observed at 75 mm stand distance with a 10 mL/min feedstock rate.

Further, challenges in the coating method, such as anodization and plasma spray technique, include adhesive strength, amorphous structure, cracks, and porosity. To overcome these challenges in the anodization and PST, post-annealing is essentially required, and it helps to improve the adhesion strength and reduce the coating defects (Zhao et al., 2021). Meanwhile, the LSP does not recommend an additional post-annealing process, and it is used to induce compressive residual stress on the surface of the material. Additionally, the LSP method alters the surface and refines the grains. Further, there is an absence of challenges such as peeling (coating layer), adhesion strength, and delamination. LSP controls the toxic elements leaching during the tribological activities due to the influence of the hardness factors (Vogt and Proriol Serre, 2021), which helps to reduce the degradation of materials (Narayanan et al., 2024). On the other hand, the laser texture method is used for the complex geometrical gyroid structure (Ti6Al4V alloy) to evaluate the impact of laser on the compression, corrosion, and metabolic activity (cytotoxicity test). The laser-treated material has not shown a negative impact in the compression (Before laser ∼93 ± 2 MPa and after laser 89 ± 3 MPa) and corrosion test. Meanwhile, the laser-treated Ti6Al4V alloy response 70% higher than that of the untreated sample in the cytotoxicity test. However, there is a gap to fill in the laser peening for enhancing the mechanical properties over the as-printed conditions (Školáková et al., 2025). Additionally, performing the laser peening in the infill part of the gyroid is a huge challenge. Therefore, the hybrid surface modification process is implemented on the substrate, where the hydrothermal treatment is performed on the laser-textured Ti6Al4V alloy. The study concluded that the corrosion, tribo-corrosion, and biocompatible properties of the material show outstanding results compared to the laser-textured and untreated samples. The reason behind the impressive response in a cytocompatibility study is due to the formation of the nanostructured oxide layer and metastable anatase dispersed barrier layer (Gupta et al., 2025).

From the knowledge of previous studies, clinical studies did not emphasize clearly that plasma-sprayed coating would enhance the life span of a patient compared to uncoated implants. A load-bearing joint subjected to dynamic motion leads to the formation of wear debris. This debris usually forms when a material is subjected to friction between joints, and it induces physical pain with inflammation at the implanted location. To alter the material’s surface property against wear and corrosion, surface modification techniques like acid etching, sandblasting, anodizing, nitriding, and coating methods (plasma spraying, pulsed laser deposition, pulsed vapor deposition, electrophoretic deposition, and sputter deposition) are frequently employed. Implanting calcium and phosphorus can help titanium alloys become more biocompatible. Conversely, nitrogen ion implantation guards the titanium surface against wear, while silver ion implantation is employed for antibacterial purposes. To fully understand the bone responses with coated-implant surfaces, comprehensive animal tests and clinical trials are necessary for future research. These ion implantations are crucial to evaluate in the future because other ions, such as strontium and silicon, are also claimed to enhance osteoconductive characteristics.

### 3.1 Future scope

The coating technologies show their performance in the mechanical and biological responses are good when compared with the untreated material surface. However, the challenges in the coating technology, such as spalling, cracks, porosity, and thermal stability, need to be controlled and are related to the coating parameters. Deep learning could be a choice to predict the coating performance, the defects, and enhance the hardness. Further, the LSP and laser texture need more attention in the future because of their unique process methods, which enhance the surface properties and biological response, which could be an optimum method for biomedical applications.

## 4 Conclusion

In conclusion, the successful integration of bio-implants into the human body is critically dependent on a range of surface modification and post-processing techniques. As this review has demonstrated, techniques such as ion implantation, anodization, and plasma spraying effectively enhance surface properties like hardness, wear resistance, and biocompatibility by creating specific coatings. Among the coating technologies, the plasma spray technique has been cleared in clinical experiments and used in hip stems. Furthermore, advanced post-processing methods like laser shock peening and surface texturing play a crucial role in improving the mechanical and tribological performance of implants, ensuring long-term durability and osseointegration. Additionally, it's in the clinical experimental, and it may be an option for the biomedical application in the future. The ongoing evolution of these methods, including the use of hybrid approaches and composite coatings, underscores a growing understanding that a tailored surface is essential for optimizing implant function and patient outcomes. Ultimately, the future of biomedical implants lies in a multi-faceted approach to material surface engineering that harmonizes mechanical integrity with biological response.
